# Pulmonary Congestion and Anemia in Hemodialysis: The Potential Link to Inflammation

**DOI:** 10.3390/ijms252011263

**Published:** 2024-10-19

**Authors:** Saleh Kaysi, Bakhtar Pacha, Marie-Hélène Antoine, Eric De Prez, Joëlle Nortier

**Affiliations:** 1 Nephrology Department, Brugmann University Hospital, Université libre de Bruxelles, 4 place Van Gehuchten, 1020 Brussels, Belgium; saleh.kaysi@chu-brugmann.be (S.K.);; 2 Laboratory of Experimental Nephrology, Faculty of Medicine, Université libre de Bruxelles, Erasme Campus, 808 route de Lennik, 1070 Brussels, Belgium

**Keywords:** pulmonary congestion, hemodialysis, dry weight, volume management, inflammation, anemia, suPAR, sST2

## Abstract

Pulmonary congestion (PC) is common in hemodialysis (HD) patients. We explored the association of anemia and pulmonary congestion in HD patients. A prospective pilot observational study included 18 patients on maintenance HD. Individual B-lines scores (BLS; 8-sites method) were obtained by lung ultrasound, before and after the first two consecutive HD sessions of the week (HD1-HD2), with different inter-dialytic intervals (68 vs. 44 h). Bioimpedance spectroscopy body composition (BIS) was performed before each HD session. Hemoglobin (Hb) levels, in addition to circulating markers of chronic inflammation (soluble urokinase Plasminogen Activator Receptor [suPAR], soluble Suppression of Tumorigenicity 2 [sST2]) were obtained. Mean (±SD) BLS values were quite elevated at all time points: Pre-HD1 (16 ± 5.53), post-HD1 (15.3 ± 6.63), pre-HD2 (16.3 ± 5.26) and post-HD2 (13.6 ± 5.83), respectively. No direct significant correlation was found between inflammation markers levels and BLS. However, mean levels (±SD, ng/mL) of suPAR pre-HD1 (7.88 ± 3.07) and pre-HD2 (7.78 ± 3.02) remained significantly above the normal range (<4 ng/mL), and sST2 levels reached 2-fold the upper normal value in most patients (27.4 ± 17.8). Pulmonary congestion reflected by BLS was negatively correlated to Hb levels pre-HD1 (R² = 0.439, *p* = 0.003), and pre-HD2 (R² = 0.301, *p* = 0.018). In addition, Hb levels were negatively correlated to global volume status estimated by BIS (R² = 0.351, *p* = 0.009). Hemoglobin levels were negatively correlated to pulmonary congestion and to the global volume status evaluated by BIS. Chronic inflammation markers were increased in HD patients, suggesting a complex volume- and non-volume-dependent pathophysiology of pulmonary congestion in HD patients.

## 1. Introduction

Pulmonary congestion (PC) is a common feature in end-stage kidney disease (ESKD) patients treated with hemodialysis (HD). Its assessment is important as it contributes to morbidity and mortality in this population.

Lung ultrasound (LUS) has proven to be an excellent and reliable tool to detect pulmonary congestion [1,2,3,4]. It was shown that PC assessed by a validated B-lines score (BLS) using LUS is common among asymptomatic HD and peritoneal dialysis patients [5].

In patients on maintenance HD, regardless of volume overload, PC is associated with adverse outcomes [5,6,7,8] and is an independent risk factor for death and cardiovascular events [9].

The pathophysiology of pulmonary congestion is complex in HD patients and includes volume- and non-volume-dependent factors. Ultrafiltration during the HD session eliminates the volume overload that is progressively accumulated between two consecutive dialysis sessions, as HD patients cannot rely on their reduced urine output to regulate their volume status.

Pulmonary congestion in HD patients may be the result of volume accumulation, cardiac dysfunction, or lung alveolar-capillary barrier permeability perturbation. The first two etiologies may be referred to as cardiogenic pulmonary congestion. Low urine output in these patients produces fluid accumulation between dialysis sessions, which increases the pulmonary capillary pressure and results in PC. It is also well known that cardiac dysfunction is common in HD patients, leading to increased pulmonary capillary pressure and pulmonary congestion. On the other hand, increased lung permeability in HD patients produces what could be referred to as non-cardiogenic pulmonary congestion. This abnormal permeability is probably due to inflammatory, toxic, and oxidative components. Some studies explored this by referring to non-volume-related increases in interstitial lung water as the so-called “uremic lung” [10,11,12].

Anemia is a well-known complication of ESKD, and it is common in HD patients. Inflammation and uremic toxins may have a negative impact on Hb levels and make anemia difficult or even resistant to treatment [13,14].

In our previous study, we found a weak correlation between PC assessed by BLS and global volume status estimated by Bioimpedance spectroscopy (BIS). Furthermore, no correlation was found between PC and systemic congestion estimated by IVC dimensions. These findings suggest a complex pathophysiology of PC in HD patients including volume- and non-volume-dependent factors [15].

Lower Hb levels in HD patients might be related to a chronic inflammatory state. The latter may also be involved in the pathophysiology of PC. We then hypothesize that a patient with a lower Hb level will probably have a higher degree of pulmonary congestion, both related to chronic inflammation and uremia.

Therefore, in the present study, we aimed to explore the potential role of chronic systemic inflammation in the pathophysiology of pulmonary congestion and its correlation to anemia.

## 2. Results

Baseline characteristics of the study population are summarized in Table 1. Most patients were hypertensive men and one-third had type 2 diabetes. Only three patients were diagnosed with heart failure. Nutrition parameters and dialysis efficacy criteria were in the target range.

Mean BLS (±SD) before and after the first HD session was high (16 ± 5.53 vs. 15.3 ± 6.63), as in the second HD session (16.2 ± 5.26 vs. 13.6 ± 5.83), respectively.

Ultrafiltration reduced BLS and this effect was more obvious in the second dialysis session (*p* = 0.03); however, BLS remained relatively high after the session despite its reduction by the UF.

### 2.1. Serum Levels of Systemic Chronic Inflammation Markers

**Figure 1:**
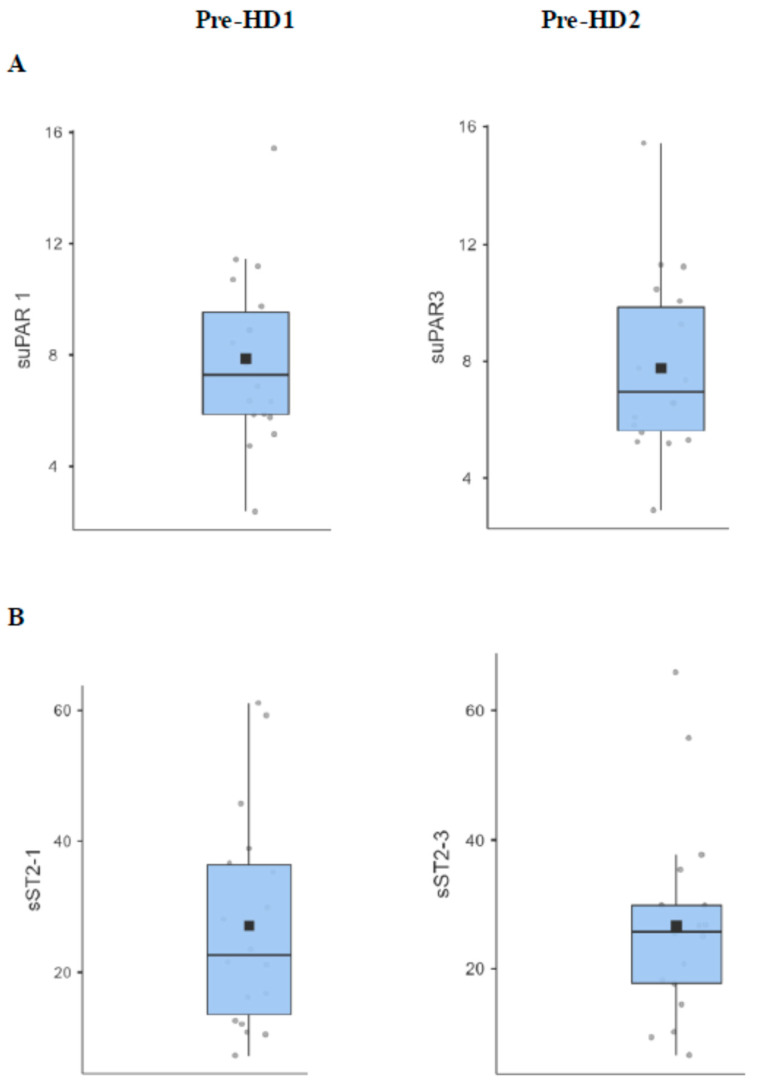
Serum pre-HD levels (in ng/mL) of suPAR (**A**) and sST2 (**B**) at different time points of the observational study (pre-HD1 and pre-HD2, respectively). suPAR: soluble urokinase Plasminogen Activator Receptor; sST2: soluble Suppression of Tumorigenicity 2; HD: Hemodialysis.

Mean serum levels of suPAR measured at different time points of the study were largely variable from one patient to another but remained significantly above the normal range (<4 ng/mL) (Figure 1A).

In parallel, mean serum sST2 levels reached 2-fold the upper normal value in most patients (Figure 1B).

### 2.2. Hemoglobin Levels and Pulmonary Congestion

**Figure 2:**
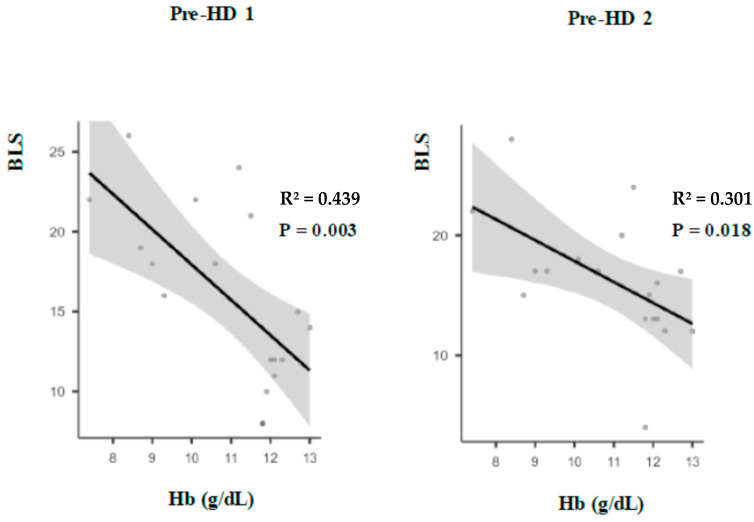
Correlations between individual serum hemoglobin levels in (g/dL) and BLS assessed pulmonary congestion before HD session 1 (pre-HD1) and 2 (pre-HD2), respectively. BLS: B lines score; Hb: Hemoglobin; HD: Hemodialysis. This negative correlation shows that a lower level of hemoglobin is correlated to a greater pulmonary congestion level. This correlation cannot only be explained by hypervolemia as the correlation between BLS and global volume status was not significant.

Pulmonary congestion reflected by BLS was negatively correlated to Hb levels pre-HD1 (R² = 0.439, *p* = 0.003), and pre-HD2 (R² = 0.301, *p* = 0.018) (Figure 2).

### 2.3. Hemoglobin Levels and Volume Markers

In addition, serum Hb levels were negatively correlated to the global volume status estimated by BIS (R² = 0.351, *p* = 0.009). However, no significant correlation was found between pulmonary congestion and BIS, although hemoglobin level was negatively correlated to pulmonary congestion, meaning that the correlation between hemoglobin and pulmonary congestion cannot be explained only by hypervolemia.

## 3. Discussion

Our study examined patients on maintenance HD and found that PC detected by LUS was quite prevalent. Although BLS was lower after both dialysis sessions, indicating that UF was effective in lowering BLS, it remained relatively high. This goes in line with the work of Noble et al., who had demonstrated that UF induced a concomitant reduction in B lines during dialysis treatment [16].

In addition, we showed that BLS levels did not significantly vary according to different inter-dialytic time intervals, suggesting that factors other than fluid accumulation might play a role in the etiology of PC in HD patients.

Among these factors, volume redistribution through a damaged alveolar-capillary barrier may explain why PC estimated by LUS is not always related to volume overload. Supporting our findings, two studies found that BLS measured with total body water by bioelectrical impedance analysis (BIA) were very weakly associated [17,18]. This structure damage might be the result of inflammation, oxidative stress, or other causes related to uremia. This hypothetical mechanism is supported by the high suPAR and sST2 serum levels that we found in almost all patients, reflecting a chronic inflammatory state in this population.

Many studies have shown that the degree of PC is a predictor of mortality and cardiovascular events [5,16]. We hypothesize that the worse prognosis related to a high BLS is the result of multiple factors, such as volume overload, cardiac dysfunction, and less respiratory capacity, in addition to inflammatory and toxic elements affecting the lungs and other organs.

A chronic inflammatory state is established when active inflammation fails to resolve [19]. In contrast to localized inflammatory responses, systemic chronic inflammation (SCI) is a state of persistent, low-grade immune activation affecting multiple physiological systems. No clear definition has been established for the state of SCI. The current standard for indicating its presence is slightly elevated C-reactive protein (CRP) levels (>3 mg/L) measured with high-sensitivity tests [20,21]. However, these inflammatory markers indeed have some limitations, but until now, we do not have better alternatives. New markers are emerging in this field, such as suPAR, which is positively correlated to several inflammatory biomarkers, including CRP, Interleukin-6 (IL-6), and Tumor Necrosis Factor-α (TNFα) [22,23,24].

CRP and suPAR reflect different aspects of inflammation. While CRP is a marker of acute infection and metabolic inflammation, suPAR is a marker of cellular inflammation and subclinical organ damage [25]. Among acute trauma patients, suPAR measured shortly after trauma was not associated with the severity of the trauma but was higher in those who later died compared with those who survived. Thus, an acute event might not immediately affect the suPAR level, but the basal suPAR level at the time of the event reflects the level of SCI, which is associated with the outcome. In other words, suPAR reflects a more chronic aspect of inflammation [26,27]. In critically ill patients, elevated or increasing suPAR levels were observed in non-survivors from the time of admission, contrasting with stable or decreased levels measured in survivors until discharge [28]. It should be underlined that suPAR is removed from the circulation via renal excretion [29]. However, suPAR retains its prognostic value even at low glomerular filtration rates [30], as it has been considered a marker of kidney disease, sepsis, cardiovascular disease, and inflammatory disorders [31,32,33,34]. The level of suPAR has been demonstrated to provide prognostic data concerning the risk of cardiovascular events and all-cause mortality in the general population and critically ill patients [23,35,36].

On the other hand, circulating sST2 levels increase in response to inflammatory diseases and heart diseases, and correlations between sST2 and inflammatory and cardiac biomarkers (CRP, NT-proBNP) were found to be significant [37]. Levels of sST2 were higher in CKD patients compared to healthy controls and were correlated with disease severity [38]. These results agree with our findings in the present study of HD patients.

Finally, we found that PC was negatively correlated to Hb levels. It was previously shown that fluid status, as defined by the overhydration level measured with bioimpedance, was negatively correlated to Hb concentrations in non-dialysis CKD [39]. In our HD patients, a similar negative correlation between Hb levels and global volume status estimated by BIS was found.

Anemia in CKD is multifactorial. A low Hb concentration may be the result of a reduced red blood cell volume and/or an increased extracellular water (ECW) volume leading to hemodilution [40,41,42]. Volume overload is strongly associated with both traditional and non-traditional risk factors for CKD progression and cardiovascular disease in CKD patients [43]. Building on that, patients with anemia may have a higher risk due to lower Hb levels and/or volume overload manifested by hemodilution.

Indeed, it was shown that anemia with volume overload was associated with a higher risk of cardiovascular morbidity and mortality as compared with anemia without volume overload. Furthermore, anemic patients with volume overload had significantly higher levels of IL-6 compared with those without volume overload [39]. In addition, a secondary analysis of the Trial to Reduce Cardiovascular Events with Aranesp Therapy (TREAT) showed that patients with a poor response to darbepoetin alfa had an increased risk of cardiovascular outcomes [44]. Consequently, serum Hb concentration may be considered as a marker for poor prognosis, indicating SCI reflected by erythropoietin treatment resistance. This inflammatory state in ESKD may contribute to the pathophysiology of PC, explaining at least in part the correlation between anemia and BLS found in our HD patients, keeping in mind that the correlation between global volume status estimated by BIS and PC was weak [45].

In conclusion, PC is common in HD patients and is inversely correlated to Hb levels. In addition, a chronic inflammatory state is common in HD patients, supporting a complex pathophysiological origin of pulmonary congestion in these patients, which should be regarded as the result of volume- and non-volume-dependent factors.

Our study has limitations; it is a pilot study with a small sample size and a high ratio of diabetic patients, in addition to the lack of assessment of CRP, IL-6, and iron status. More research should be conducted in this field to better understand the impact of inflammation on anemia and lung congestion in chronic kidney disease.

## 4. Materials and Methods

**Figure 3:**
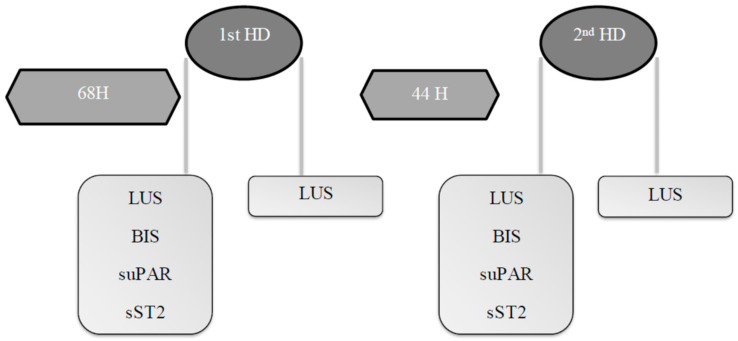
Schematic time course and design of the pilot observational study. LUS: Lung ultrasound; BIS: Bioimpedance spectroscopy; HD: Hemodialysis; suPAR: soluble urokinase Plasminogen Activator Receptor; sST2: soluble Suppression of Tumorigenicity 2.

The study design was previously described [15] and is briefly summarized in Figure 3. All participants were recruited from our HD unit at Brugmann University Hospital.

The study received approval from the Ethics Committee of our hospital and was performed according to institutional procedures and the Declaration of Helsinki. All participants signed a written informed consent before inclusion.

Plasma samples collected during our previous prospective observational study were used to measure the concentration of circulating markers of chronic systemic inflammation such as soluble urokinase Plasminogen Activator Receptor [suPAR] and soluble Suppression of Tumorigenicity 2 [sST2].

### 4.1. Patient Inclusion/Exclusion Criteria and Clinical/Biological Data Collection

Eighteen adult patients on maintenance HD for at least 3 months in our high-care unit were included. Patients diagnosed with interstitial lung disease, recent pneumonia, previous lung surgery, or those who had cancer were excluded.

### 4.2. Design of the Protocol

Charts with the most up-to-date values available were reviewed to collect data, including demographics (age, gender), HD treatment parameters, cause of ESKD (clinically suspected or biopsy-proven), laboratory parameters (serum urea, albumin, Hb), dry weight (DW), weight before and after HD sessions, pre- and post-dialysis blood pressure, in addition to comorbidities such as diabetes, previous cardiovascular events, and treatment with antihypertensive agents.

All patients underwent LUS in a supine or near-supine position before and after their regularly scheduled first and second sessions of the week. All measurements were performed by the same operator at the bedside, using the same ultrasound machine (T-Lite system, Sonoscanner, Meditor, France).

To quantify PC, an individual B-lines score (BLS) was obtained according to the 8-sites method. A global BLS of more than 5 was considered to reflect moderate to severe PC [46].

Circulating markers of inflammation (suPAR and sST2) were also measured before each HD session

In addition, bioimpedance spectroscopy (BIS) was performed before each HD session using a portable whole-body bio-impedance spectroscopy device (BCM, Fresenius Medical Care D GmbH, Germany).

### 4.3. Statistics

Statistical analyses were performed using Jamovi software(https://www.jamovi.org/). Continuous variables were expressed as means ± standard deviation (SD), or median (interquartile range) as appropriate, and categorical variables as absolute and relevant frequencies.

Comparisons of continuous variables were performed with the paired-samples Student *t*-test or Wilcoxon’s signed rank test and with the Student *t*-test for independent samples or the Mann–Whitney test, according to the normality of the distribution. Categorical variables were compared using the x2 test or Fisher exact test.

## Figures and Tables

**Table 1:** Patients’ baseline clinical and biological characteristics.

Variable	Value
Number	18
Mean age (years) (min–max)	68 (24–88)
Female/Male ratio	3/15
Diabetics, *n* (%)	6 (33%)
Hypertension, *n* (%)	16 (89%)
Heart failure, *n* (%)	3 (17%)
AVF, *n* (%)	10 (55%)
Central hemocatheter, *n* (%)	8 (45%)
HD, *n* (%)	8 (45%)
HDF, *n* (%)	10 (55%)
Mean BMI, kg/m² (min–max)	25.6 (15–34)
Mean hemoglobin in g/dl (min–max)	10.8 (7.4–12.7)
Mean Kt/V (±SD)	1.74 (±0.38)
Mean dialysis vintage (months)	65.9 (±60)
Residual urine volume (mL/24 h)	437 (±300)
Mean albuminemia (g/dL) (±SD)	40.6 (±4.1)
Mean calcemia (mmol/L) (±SD)	2.36 (±0.2)
Mean kalemia (mmol/L) (±SD)	5.0 (±1.1)
Mean phosphoremia (mmol/L) (±SD)	1.53 (±0.2)
Mean pre-dialysis urea (mg/dL) (±SD)	123 (±18)
Mean post-dialysis urea (mg/dL) (±SD)	29.4 (±10)

AVF: Arteriovenous Fistula; BMI: Body Mass Index; HD: Hemodialysis; HDF: Hemodiafiltration.

## Data Availability

All data are available online: https://doi.org/10.6084/m9.figshare.24099672.
